# Healthy volunteers in US phase I clinical trials: Sociodemographic characteristics and participation over time

**DOI:** 10.1371/journal.pone.0256994

**Published:** 2021-09-07

**Authors:** Corey A. Kalbaugh, Julianne M. Kalbaugh, Lisa McManus, Jill A. Fisher

**Affiliations:** 1 Department of Public Health Sciences, Clemson University, Clemson, SC, United States of America; 2 Department of Bioengineering, Clemson University, Clemson, SC, United States of America; 3 Department of Social Medicine and Center for Bioethics, School of Medicine, The University of North Carolina at Chapel Hill, Chapel Hill, NC, United States of America; 4 Department of Sociology, Wake Technical Community College, Raleigh, NC, United States of America; Oregon State University, UNITED STATES

## Abstract

**Background:**

Increasing the diversity of research participants is an important focus of clinical trials. However, little is known regarding who enrolls as healthy volunteers in Phase I clinical trials, which test the safety and tolerability of investigational new drugs. Despite the risk, healthy volunteers can derive no medical benefit from their participation, and they are financially compensated for enrolling.

**Objective:**

This study’s purpose is to describe sociodemographic characteristics and clinical trial participation histories of healthy people who enroll in US Phase I trials.

**Methods:**

The HealthyVOICES Project (HVP) is a longitudinal study of healthy individuals who have enrolled in Phase I trials. We describe self-reported sociodemographic information and Phase I trial history from HVP recruitment (May-December 2013) through the project’s end three years later (December 2016). Trial experiences are presented as medians and quartiles.

**Results:**

The HVP included 178 participants. Nearly three-fourths of participants were male, and two-thirds were classified as racial and ethnic minorities. We found that some groups of participants were more likely to have completed a greater number of clinical trials over a longer timeframe than others. Those groups included participants who were male, Black, Hispanic, 30-39-years-old, unemployed, had received vocational training in a trade, or had annual household incomes of less than $25,000. Additionally, the greater the number of clinical trials participants had completed, the more likely they were to continue screening for new trials over the course of three years. Participants who pursued clinical trials as a full-time job participated in the greatest number of trials and were the most likely to continuing screening over time.

**Implications:**

Participation as a healthy volunteer in US Phase I trials is driven by social inequalities. Disadvantaged groups tend to participate in a greater number of clinical trials and participate longer than more privileged groups.

## Introduction

People from disadvantaged sociodemographic groups, especially people of color, are more likely to participate as healthy volunteers in US Phase I clinical trials that test drug toxicity levels and side effects [1–3]. Conversely, the same sociodemographic groups who serve as healthy volunteers in these safety trials are often underrepresented in later-stage trials where some therapeutic benefit could be derived [4–6]. Additionally, unlike the improvements that have been made in later-phase trials, women continue to be underrepresented as healthy volunteers in Phase I trials [6]. This misaligned representation in clinical trial research is troubling for reasons of equity and justice that warrant further examination [7–9].

Research has also found that healthy volunteers are often not simply one-time participants but are “serial” participants who are likely to enroll repeatedly in Phase I trials [10–12]. This is largely assumed to be associated with the financial compensation that healthy volunteers receive for their trial participation [13–15], and some healthy volunteers even become “professional guinea pigs” [16, 17]. To date, however, few studies have explored how participation might differ based on healthy volunteers’ sociodemographic characteristics such as race and ethnicity, sex, educational attainment, employment status, and household income [2, 18]. Even less is known about healthy volunteers’ participation in clinical trials over time. Groups that participate serially in trials could be exposing themselves to additional risk, which could be interpreted as a form of exploitation in medical research, particularly if this burden falls disproportionately on people of color and other disadvantaged groups.

The purpose of this study is to describe the patterns of healthy volunteers’ Phase I participation in the US, including the number of clinical trials in which they have enrolled and the number of years since enrolling in their first clinical trial, as well as their continued enrollment in or attrition from trials over a three-year period. Having a clearer picture of healthy volunteers’ sociodemographic characteristics can reveal how financially motivated involvement in Phase I trials intersects with broader social inequalities.

## Methods

We present data collected during the HealthyVOICES Project (HVP), a longitudinal, mixed-methods study of healthy individuals who participated in at least one Phase I clinical trial. The overall purpose of the HVP was to better understand the perceptions of trial risks and benefits among people who enroll as healthy volunteers in clinical trials, including first-in-human trials, bioequivalence trials, and drug-drug interaction trials, while also attending to their health behaviors and decision making about clinical trials over time. More detailed descriptions of our study methods have been previously published [19, 20]. The study was reviewed and approved by the Biomedical Institutional Review Board at the University of North Carolina at Chapel Hill (#13–1256). All participants provided written informed consent.

### The healthy voices project

Participants were identified and recruited from one of seven Phase I research clinics in the United States from May to December 2013. The clinics were chosen for geographic diversity and included three sites in the Eastern US, two sites in the Midwestern US, and two sites in the Western US. Clinic types included one academic site, one pharmaceutical company site, one privately owned commercial site, and four contract research organization sites. At the time we recruited participants, the clinics were conducting heathy volunteer Phase I trials on a broad spectrum of investigational drugs including, but not limited to, cholesterol, pain, autoimmune diseases, cancer, blood-related diseases, and psychiatric illnesses.

The clinics gave permission for our research team to enroll healthy volunteers onsite, but they were not otherwise involved in the design or execution of our study. All healthy volunteers who were fluent in English or Spanish were eligible to participate. A member of the HVP team invited healthy volunteers to join the study and met one-on-one with interested individuals to discuss material contained in the HVP consent form, answer their questions, and request written consent. About 10% of the healthy volunteers invited to learn more about the HVP study declined, and one individual declined to participate after reading the consent form and discussing the study with a team member.

After providing written consent, participants completed a “baseline” enrollment survey where they self-reported their demographic information, including their sex, race, ethnicity, date of birth, employment status, educational attainment, and household income. We used the US Office of Management and Budget (OMB) standards on race and ethnicity to collect data on each category separately. For race, participants were given the option to select from the following: American Indian or Alaskan Native; Asian or Asian American; Black or African American; Native Hawaiian or Other Pacific Islander; White; or More than One Race. To report on ethnicity, participants selected between Hispanic/Latino and Not Hispanic/Latino. Participants self-reported their employment status as employed full-time, part-time, self-employed, retired, or unemployed, as well as filled in an open-response field for their current job. Because of variability in how participants interpreted and used the “self-employed” category, we used the current job field to consolidate the employment status data into three categories: “Full-time, including business owners,” “Part-time, including seasonal and gig work,” and “Not employed or retired.” Data about employment status were again collected at the end of each participant’s involvement in the HVP three years later. Educational attainment was also based on self-report at baseline and at the conclusion of the study, using the following 7 categories: Less than High School; High School/GED; Some College; Trade or Vocational Training; Associate’s (2-year) Degree; Bachelor’s (4-year) Degree; and Graduate Degree. For analytic purposes, we consolidated the responses of High School/GED and Some College into one single category of “No more than high school degree (including some college),” and we consolidated Bachelor’s (4-year) Degree and Graduate Degree into “Bachelor’s (4-year) degree or higher.” Household income was collected from participants at baseline and at the end of the study using the following categories: Less than $10,000; $10,000–$24,999; $25,000–$49,999; $50,000–$74,999; $75,000–$99,999; and ≥ $100,000. For our analysis, we used only three categories to compare groups: Less than $25,000; $25,000–$50,000; and More than $50,000. Finally, we categorized participants as “occupational” participants if they reported that clinical trials were their full-time job.

### Measurement of phase I clinical trial experience

The baseline survey had additional questions to capture participants’ clinical trial history including: the total number of Phase I studies completed (counting the one they were participating in when recruited to our study), the year they enrolled in their first Phase I trial, and estimated total earnings from their Phase I participation. We calculated the number of years they had been participating by subtracting the year of their first trial from the recruitment year (i.e., 2013). We examined the distribution of each component of clinical trial history and identified the median and interquartile range, which were then used as a cutpoint for each measure of trial experience. Results regarding trial earnings have been published elsewhere [15].

### Measurement of clinical trial activity during HVP

As part of their 3-year involvement in the HVP, participants were not required to continue to enroll in new clinical trials, but they were asked to report information about any clinical trials for which they screened. The majority of participants (79%) provided this information to the study team in real-time throughout the project using an online or telephone survey, and the remainder of the participants provided a tally of their 3-year participation as part of their final study visit. No differences in reported clinical trial participation were found between the groups [15]. More detailed information about the survey instrument and our methods to collect ongoing clinical trial participation have been previously published [15, 20].

## Results

### Study population

One-hundred seventy-eight healthy volunteers were included in the HVP, and 166 (93.3%) were retained and completed the study three years after their enrollment. The majority of participants were male (74%; Table 1), and most participants self-identified as either Black/African American (n = 72, 41%) or White (n = 83; 47%). Thirty-eight (21%) identified as Hispanic, including participants who self-identified as White, Black, more than one race, American Indian, and Native Hawaiian/Pacific Islander. Only 57 (32%) participants identified as non-Hispanic White, and two-thirds (n = 121) of participants were classified as racial/ethnic minorities. The median participant age was 39 (range: 18–64; IQR: 30, 48). At baseline, only 37 (21%) participants had a 4-year college degree (n = 32) or a graduate degree (n = 5). Over the course of three years, 13 of the 166 retained participants (8%) completed additional education, including 5 who received a 4-year college degree, one who received a 2-year college degree, and 7 who completed training in a trade. At baseline, 73 (41%) participants were unemployed or retired, another 60 (34%) worked part-time, and only 45 (25%) held full-time positions. On the whole, participants’ employment situations improved over the course of the HVP, with nearly half (46%) reporting full-time work and only about a quarter reporting being unemployed three years later. At baseline, nearly half of the participants in our study had a household income of less than $25,000. Over the course of three years, 54% of the retained participants reported the same household income bracket as baseline, 35% reported more annual income, and 11% reported less annual income.

**Table 1:** Characteristics of HVP study participants (enrolled v. retained).

	Baseline (n = 178)	3-years later (n = 166)
	N (%)	N (%)
**Sex, % female**		
**Age group**	47 (26)	45 (27)
18–29	40 (22)	23 (14)
30–39	58 (33)	45 (27)
40–49	54 (30)	51 (31)
50+	26 (15)	47 (28)
**Race/ethnicity** ^**a**^		
Asian or Asian American	6 (3)	6 (4)
American Indian or Alaskan Native	2 (1)	2 (1)
Black or African American	72 (41)	66 (40)
Native Hawaiian or Pacific Islander	2 (1)	2 (1)
White	83 (47)	80 (48)
More than one race	13 (7)	10 (6)
Hispanic	38 (21)	33 (20)
**Racial/ethnic minority, %**		
**Educational attainment**	121 (68)	111 (67)
Less than high school	12 (7)	12 (7)
High school/GED	37 (21)	29 (18)
Some college	52 (29)	40 (24)
Trade or vocational training	19 (11)	23 (14)
Associate’s (2-year) degree	21 (12)	20 (12)
Bachelor’s (4-year) degree	32 (18)	37 (22)
Graduate degree	5 (3)	5 (3)
**Employment status** ^**b**^		
Full-time, including business owners	45 (25)	76 (46)
Part-time, including seasonal & gig work	60 (34)	47 (28)
Not employed or retired	73 (41)	43 (26)
**Household income** ^**c**^		
Less than $10,000	30 (17)	20 (12)
$10,000–$24,999	52 (29)	36 (22)
$25,000–$49,999	71 (40)	64 (39)
$50,000–$74,999	13 (7)	26 (16)
$75,000–$99,999	7 (4)	6 (4)
≥ $100,000	4 (2)	11 (7)

^a^ The category Hispanic includes all racial groups, of which we have those in our sample who identified as White, Black, more than one race, American Indian, and Native Hawaiian/Pacific Islander.

^b^ These data are based on consolidated definitions of each employment category that we used to standardize self-reported data from participants.

^c^ Household income was not reported by one participant at baseline and three at the end of the HVP.

### Phase I trial experience

All participants were enrolled in a Phase I trial when we recruited them, and it was the first clinical trial for only 38 individuals (21%). Approximately one-quarter had previously enrolled in more than 10 trials (Table 2). Two-thirds of our participants had started enrolling in Phase I trials in the prior 4 years. The median number of years of participation was 3 years (IQR: 0, 7), and participants had enrolled in a median of 5 trials (IQR: 2, 12) at baseline regardless of when they first enrolled. Over the three years of the HVP, participants completed a median of 3 new clinical trials (IQR: 1, 7). Table 3 provides the medians and interquartile ranges by demographic groups for number of years since first clinical trial, the number of clinical trials at baseline, and the number of new clinical trials at the end of the HVP.

**Table 2:** Characterization of participants’ phase I trial experience.

	N (%)
**Years of participation at baseline (n = 178)**	
<1	53 (30)
1–4	65 (36)
5–9	26 (15)
≥10	34 (19)
**Number of trials at baseline (n = 178)**	
1	38 (21)
2–4	49 (28)
5–10	45 (25)
>10	46 (26)
**Number of “occupational” participants at baseline (n = 178)**	62 (35)
**Number of “occupational” participants 3 years later (n = 166)**	28 (17)
**Number of new trials 3 years later (n = 166)**	
None	37 (22)
1	19 (11)
2–4	45 (27)
5–10	50 (30)
>10	15 (9)
**Total number of trials 3 years later (n = 166)**	
1	18 (11)
2–4	30 (18)
5–10	38 (23)
11–20	36 (22)
>20	44 (27)

**Table 3:** Clinical trial activity by demographic group.

Demographic groups	Years since 1^st^ trial at baseline (n = 178) Median (IQR)	Number of trials at baseline (n = 178) Median (IQR)	Number of new trials 3 years later (n = 166) Median (IQR)
**Overall**	3 (0, 7)	5 (2, 12)	3 (1, 7)
**Sex**			
Female	2 (0, 5)	3 (2, 8)	2 (0, 5.5)
Male	3 (0, 8)	6 (2, 15)	4 (1, 7)
**Race/ethnicity**			
Black	3 (0, 8)	6 (2, 15)	4 (1, 8)
Hispanic	2 (0, 4)	4.5 (1, 9)	2 (1, 6.5)
Non-Hispanic White	2 (0, 8)	3 (1.5, 12)	2 (0, 5)
**Age**			
18–29	1 (0, 2)	3 (1, 7)	3 (0, 4)
30–39	3 (1, 6)	6 (3, 15)	4 (1, 7)
40–49	4 (0, 11)	5.5 (1, 15)	3 (1, 8)
50+	3.5 (0, 11)	7 (2, 15)	3 (0, 7)
**Educational attainment**			
Less than high school degree	1 (0, 3)	2 (1, 6)	3 (1, 3.5)
No more than high school degree (including some college)	2 (0, 5)	4 (1, 10)	3 (1, 7)
Trade or vocational training	4 (3, 10.5)	12 (3.5, 17.5)	4 (2, 8.5)
Associate’s (2-year) degree	2 (1, 5)	6 (2, 10)	3.5 (1, 7)
Bachelor’s (4-year) degree or higher	5 (1, 8)	5 (2, 15)	2.5 (0, 5)
**Employment status**			
Full-time, including business owners	3 (0, 8)	4 (1, 8)	2 (0, 5)
Part-time, including seasonal & gig work	3 (1, 6)	6 (3, 13.5)	3 (2, 6.5)
Not employed or retired	2 (0, 6)	4 (1, 12)	5 (1.5, 10)
**Household income**			
Less than $25,000	2 (0, 5)	3 (1, 9)	4 (1, 7)
$25,000–$49,999	4 (1, 8)	7 (3.5, 15)	3 (1, 7)
More than $50,000	2.5 (0, 6.5)	3.5 (1, 13)	2 (0, 4.5)
**Baseline “occupational” participants**			
Yes	4 (1, 9)	10 (5, 20.5)	8 (5, 11.5)
No	2 (0, 6)	3 (1, 7)	2 (0, 4)

#### Trial experience by sex

At baseline, female HVP participants had completed a median of 3 Phase I trials (IQR: 2, 8) and had participated in trials for a median of 2 years (IQR: 0, 5). In contrast, male HVP participants had completed twice the number of trials (median = 6; IQR: 2, 15) over a median of 3 years (IQR: 0, 8). Over the course of the HVP, this participation rate by sex remained consistent, with males participating in double the clinical trials as females in three years. Specifically, females completed a median of 2 new clinical trials (IQR: 0, 5.5), whereas males completed a median of 4 (IQR: 1, 7).

#### Trial experience by race and ethnicity

At baseline, Black participants had enrolled in twice the number of trials (median = 6; IQR: 2, 15) as Non-Hispanic White participants (median = 3; IQR: 1.5, 12). Hispanic participants reported having enrolled in a median of 4.5 (IQR: 1, 9) trials at baseline. Black participants also had a longer history of participation (median = 3 years; IQR: 0, 8) than both Non-Hispanic Whites (median = 2 years; IQR: 0, 8) and Hispanics (median = 2 years; IQR: 0, 4). In the three years of the HVP, Black participants enrolled in double the number of new clinical trials as the other groups, with a median of 4 trials (IQR: 1, 8), compared to Non-Hispanic White participants (median = 2 trials; IQR: 0, 5) and Hispanic participants (median = 2 trials; IQR: 1, 6.5).

#### Trial experience by age

The median number of baseline clinical trials and length of time since the first trial were different between participants who were younger than 30 and those who were 30 and older. Specifically, 18–29-year-olds had enrolled in a median of 3 trials (IQR: 1, 7) at baseline and had been participating in trials for a median of 1 year (IQR: 0, 2). In contrast, 30–39-year-olds had enrolled in 6 trials (IQR: 3, 15) over the span of 3 years (IQR: 1, 6); 40–49-year-olds had enrolled in 5.5 trials (IQR: 1, 15) over 4 years (IQR: 0, 11); and participants 50 and older had enrolled in 7 trials (IQR: 2, 15) trials over 3.5 years (IQR: 0, 11). Clinical trial enrollment during the HVP was largely the same among all age groups, though 30–39-year-olds had a higher rate of participation [18–29-year-olds: 3 trials (IQR: 0, 4); 30–39-year-olds: 4 trials (IQR: 1, 7); 40–49-year-olds: 3 trials (IQR: 1, 8); 50 and older: 3 trials (IQR: 0, 7)].

#### Trial experience by educational attainment

There was variation in the baseline median number of clinical trials in which participants had enrolled based on educational attainment. Specifically, participants with less than a high school education had enrolled in 2 trials (IQR: 1, 6); participants with no more than a high school degree (regardless of some college experience) had enrolled in 4 trials (IQR: 1, 10); participants with a trade or vocational training had enrolled in 12 trials (IQR: 3.5, 17.5); participants with an associate’s degree had enrolled in 6 trials (IQR: 2, 10); and participants with a bachelor’s degree or higher had enrolled in 5 trials (IQR: 2, 15). Some of the difference in trial numbers might be a function of time since first trial, but not exclusively. Participants with less than a high school education had been involved in trials for 1 year (IQR: 0, 3); participants with no more than a high school degree had been involved in trials for 2 years (IQR: 0, 5); participants with a trade or vocational training had been involved in trials for 4 years (IQR: 3, 10.5); participants with an associate’s degree had been involved in trials for 2 years (IQR: 1, 5); and participants with a bachelor’s degree or higher had been involved in trials for 5 years (IQR: 1, 8). Time does not seem to be the only factor to explain differences in baseline trials because participants’ clinical trial activity by educational attainment was largely mirrored in the three-year period of the HVP. Participants with a trade or vocational training completed the largest number of new clinical trials in three years (median = 4, IQR: 2, 8.5), whereas the median numbers of new clinical trials clustered around 3 for participants with all other levels of educational attainment.

#### Trial experience by employment status

Unlike the demographic categories of sex and race/ethnicity, employment status is highly dynamic and much more subject to change, even compared to educational attainment (particularly given that individuals do not lose educational attainment over time). This makes it difficult to characterize participants’ history of clinical trial participation through the snapshot of their employment at baseline and three years later. Nonetheless, individuals who were employed part-time at baseline had participated in a greater number of clinical trials (median = 6, IQR: 3, 13.5) than those who worked full time (median = 4, IQR: 1, 8) or were unemployed (median = 4, IQR: 1, 12). However, participants who were unemployed at baseline were newer to clinical trials (median = 2 years, IQR: 0, 6) relative to those who were employed part time (median = 3 years, IQR:1, 6) or worked full time (median = 3 years, IQR: 0, 8). These numbers suggest that the difference in trial numbers between part-time workers and unemployed participants might have been a function of time since first trial. Comparing the number of new clinical trials in which participants enrolled during the HVP, people who were unemployed at the end of the study completed the most trials (median = 5, IQR: 1.5, 10) followed by people who were employed part-time (median = 3, IQR: 2, 6.5), then people who worked full time (median = 2, IQR: 0, 5).

#### Trial experience by household income

As with employment status, household income as a demographic category does not lend itself as well to comparing groups over time because it can fluctuate dramatically from year to year. That said, those who earned between $25,000 and $50,000 annually had participated in the most clinical trials over the longest period of time (median = 7 trials, IQR: 3.5, 15; median = 4 years, IQR: 1, 8) compared to participant who earned less than $25,000 annually (median = 3 trials, IQR: 1, 9; median = 2 years, IQR: 0, 5) or more than $50,000 annually (median = 3.5 trials, IQR: 1, 13; median = 2.5 years, IQR: 0, 6.5). Using household income at the end of the HVP to examine study participation, those who earned less than $25,000 annually participated in a greater number of trials (median = 4, IQR: 1, 7) than those who earned between $25,000 and $50,000 annually (median = 3, IQR: 1, 7) who, in turn, participated in a greater number of trials than those who earned more than $50,000 annually (median = 2; IQR: 0, 4.5).

#### Trial experience by “occupational” participation

Sixty-two participants (35%) were categorized as “occupational” participants because they reported pursuing clinical trials as a full-time job. Occupational participants had participated in a median of 10 trials (IQR: 5, 20.5) at baseline and had been enrolling in trials for a median of 4 years (IQR: 1, 9). Over the course of the HVP, they enrolled in a median of 8 new trials (IQR: 5, 11.5). In contrast, at baseline, the non-occupational participants had enrolled in a median of 3 trials (IQR: 1, 7) and had been participating in trials for a median of 2 years (IQR: 0, 6). This group enrolled in a median of 2 new trials (IQR: 0, 4) in the 3 years of the HVP. Thus, occupational participant had enrolled in about three times the number of clinical trials at baseline, had been participating twice as long, and enrolled in four times as many new trials during the HVP. There were no meaningful demographic differences—such as by race/ethnicity, sex, or age—between the occupational participants and the rest of the HVP sample.

### Attrition from serial trial participation

Despite the norm of serial participation, many HVP participants were no longer involved in clinical trials at the end of three years. There was a steady decline in the number of participants who screened and participated in each of the three years of the HVP. From 100% participation at baseline, only 90 of the 166 retained participants (54%) had screened for any new clinical trials in the final 12 months of the HVP, and only 73 (44%) had actually enrolled in a trial that year. Table 4 provides the percentages of each demographic group that were still screening and participating in clinical trials in the final year of the HVP.

**Table 4:** Attrition from phase I participation over 3 years by demographic group (N = 166).

Demographic groups	Still screening N (% of group)	Still participating N (% of group)
**Overall**	90 (54)	73 (44)
**Sex**		
Female	25 (56)	17 (38)
Male	65 (54)	56 (46)
**Race/ethnicity**		
Black	40 (61)	31 (47)
Hispanic	17 (52)	12 (36)
Non-Hispanic White	23 (42)	21 (38)
**Age**		
18–29	11 (48)	7 (30)
30–39	24 (53)	18 (40)
40–49	27 (53)	24 (47)
50+	28 (60)	24 (51)
**Educational attainment**		
Less than high school degree	7 (58)	5 (42)
No more than high school degree (including some college)	35 (51)	29 (42)
Trade or vocational training	15 (65)	13 (57)
Associate’s (2-year) degree	12 (60)	10 (50)
Bachelor’s (4-year) degree or higher	21 (50)	16 (38)
**Employment status**		
Full-time, including business owners	28 (37)	23 (30)
Part-time, including seasonal & gig work	33 (70)	22 (47)
Not employed or retired	29 (67)	28 (65)
**Household income**		
Less than $25,000	39 (70)	30 (54)
$25,000–$49,999	30 (47)	26 (41)
More than $50,000	20 (47)	16 (37)
**Baseline “occupational” participants**		
Yes	46 (79)	40 (69)
No	44 (41)	33 (31)
**Clinical trial experience at baseline**		
1	6 (17)	4 (11)
2–4	23 (56)	20 (49)
5–10	28 (62)	22 (49)
>10	33 (73)	27 (60)
[Combined >1]	[84 (64)]	[69 (53)]

#### Attrition by sex

There was no important difference in the percentage of females and males who continued to screen for clinical trials in the third year of the HVP (56% v 54%). However, an appreciably greater percentage of males actually completed at least one clinical trial that year (46% v 38%).

#### Attrition by race and ethnicity

After three years, 61% of Black participants, 52% of Hispanic participants, and 42% of Non-Hispanic White participants continued to screen for clinical trials. While there were differences in screening rates among each of these three groups, the primary difference in trial enrollment rates was between Black participants and the others. Specifically, 47% of Black participants enrolled in at least one trial in the final year of the HVP, whereas 38% of Non-Hispanic Whites and only 36% of Hispanics did.

#### Attrition by age

There was more attrition from clinical trial screening and enrollment among younger participants. Specifically, in the final year of the HVP, 48% of 20–29-year-olds, 53% of 30–39-year-olds, 53% of 40–49-year-olds, and 60% of participants 50 and older screened for at least one clinical trial. The age-related pattern was more striking for trial enrollment with 30% of 20–29-year-olds, 40% of 30–39-year-olds, 47% of 40–49-year-olds, and 51% of participants 50 and older completing at least one new trial.

#### Attrition by educational attainment

The largest differences in attrition by educational attainment could be seen between those participants with a trade/vocational training or with an associate’s degree and all other participants. Sixty-five percent of participants with a trade were still screening, and 57% were still participating in clinical trials at the end of the HVP. Similarly, 60% of participants with an associate’s degree were still screening, and 50% were still participating. Those participants with less than a high school education were screening at comparable rates (58% of this group), but only 42% had participated in any trials in the final year of the study. Of those participants who had no more than a high school education (including some college), 51% were still screening and 42% still participating. Those participants with a bachelor’s degree or higher had the most attrition from clinical trial participation, with just 50% still screening and 38% still participating.

#### Attrition by employment status

Among the 90 HVP participants still screening for Phase I trials at the end of the study, 31% worked full time, 37% worked part time, and 32% were unemployed. The percentages shift modestly when examining only the 73 participants who actually enrolled in at least one clinical trial in the final year of the HVP when 32% worked full time, 30% worked part time, and 38% were unemployed. This shows the continued involvement of participants in clinical trials from all employment groups. Examining continued clinical trial activity by employment status, however, provides a different view of attrition. Of those participants with full-time employment at the end of the HVP, 37% were still screening and 30% were still participating, whereas 70% of part-time workers were still screening and 47% still participating. In contrast, 67% of unemployed participants were still screening and 65% had enrolled in at least one trial in the final year of the study.

#### Attrition by household income

HVP participants with a household income of less than $25,000 per year were the most likely to still be screening for studies compared to participants who earned between $25,000 and $50,000 or who earned more than $50,000 annually (70% v 47%). However, the difference among participants in these three income brackets was less pronounced when examining the percentage of participants who had actually enrolled in a trial during the final year of the HVP. Specifically, 54% of participants with an annual household income of less than $25,000, 41% of participants with an annual household income between $25,000 and $50,000, and 37% of participants with an annual household income over $50,000 enrolled in at least one trial.

#### Attrition by occupational participation

Compared to the sample as a whole, there was less attrition from clinical trial involvement among those participants who were occupational participants at baseline. Of that group, 79% were still screening and 69% were still participating in the final year of the HVP. Only 41% and 31% of those individuals who were not occupational participants at baseline were still screening and participating, respectively, at the end of the HVP. Despite the differences between these groups, it was also the case that the number of occupational participants declined from 62 at baseline to 28 at the end of the HVP.

#### Attrition by baseline clinical trial experience

The more clinical trial experience a participant had at baseline the more likely they were to still be screening and participating at the end of the HVP. Therefore, the most attrition occurred among first-time participants, of whom only 6 (17%) were still screening and only 4 (11%) were still participating. The rest of the sample combined (i.e., anyone who had participated in at least two trials at baseline) had screening and participation rates of 64% and 53% respectively. Splitting the first-time participants into the groups of “single-time” participants and those who had enrolled in at least one more trial after baseline, 18 of the 35 (51%) first-time participants retained for three years in the HVP did not enroll in any subsequent clinical trials. In examining those participants who had enrolled only a single time in a clinical trial, there were no differences based on sex or race/ethnicity between them and the other first-time participants who continued to enroll in clinical trials. However, older participants were more likely to be single-time participants. Specifically, two-thirds of first-time participants between the ages of 40–49 and those over 50 did not enroll in another clinical trial after baseline compared to only about 30% between the ages of 30–39 and 40% between the ages of 20–29. Among those first-time participants who enrolled in at least one more trial, 6 (36%) were still screening and 4 (24%) were still participating at the end of the HVP. These rates are still lower than participants who had enrolled in 2 to 4 trials at baseline of whom 56% and 49% were still screening and participating. In the group of participants who had enrolled in 5 to 10 trials at baseline, 62% and 49% were still screening and participating, respectively. In contrast, 73% and 60% of participants who had enrolled in more than 10 trials at baseline were still screening and participating, respectively.

## Discussion

**Fig 1:**
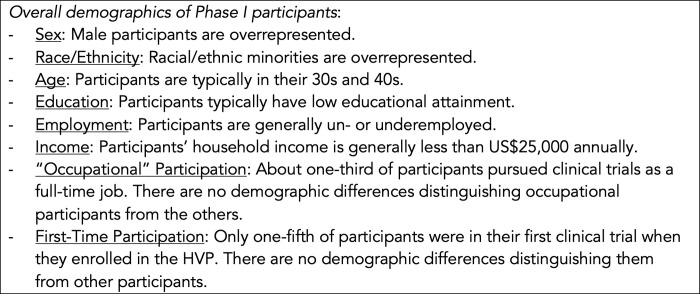
Narrative summary of demographic findings. *Overall demographics of Phase I participants*: Sex: Male participants are overrepresented. Race/Ethnicity: Racial/ethnic minorities are overrepresented. Age: Participants are typically in their 30s and 40s. Education: Participants typically have low educational attainment. Employment: Participants are generally un- or underemployed. Income: Participants’ household income is generally less than US$25,000 annually. “Occupational” Participation: About one-third of participants pursued clinical trials as a full-time job. There are no demographic differences distinguishing occupational participants from the others. First-Time Participation: Only one-fifth of participants were in their first clinical trial when they enrolled in the HVP. There are no demographic differences distinguishing them from other participants.

**Fig 2:**
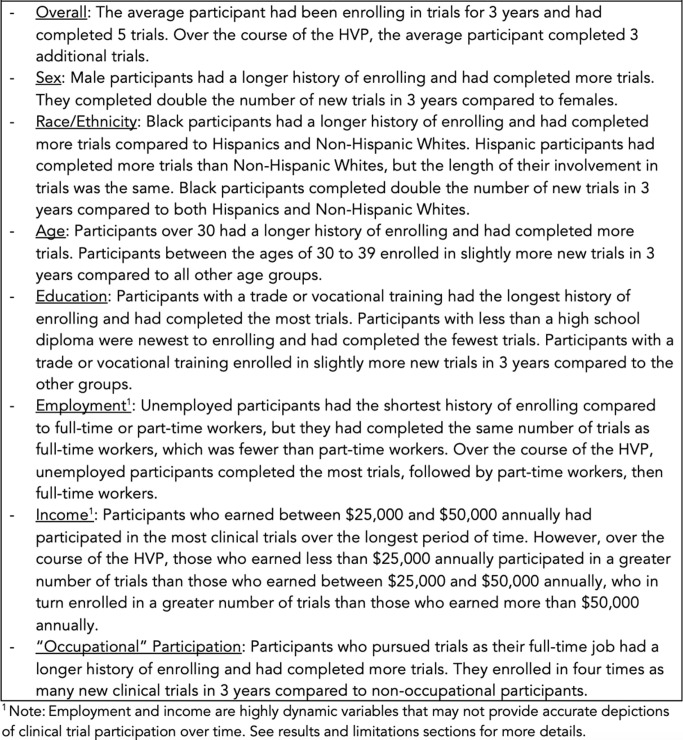
Summary of phase I trial history and experience by demographic group. Overall: The average participant had been enrolling in trials for 3 years and had completed 5 trials. Over the course of the HVP, the average participant completed 3 additional trials. Sex: Male participants had a longer history of enrolling and had completed more trials. They completed double the number of new trials in 3 years compared to females. Race/Ethnicity: Black participants had a longer history of enrolling and had completed more trials compared to Hispanics and Non-Hispanic Whites. Hispanic participants had completed more trials than Non-Hispanic Whites, but the length of their involvement in trials was the same. Black participants completed double the number of new trials in 3 years compared to both Hispanics and Non-Hispanic Whites. Age: Participants over 30 had a longer history of enrolling and had completed more trials. Participants between the ages of 30 to 39 enrolled in slightly more new trials in 3 years compared to all other age groups. Education: Participants with a trade or vocational training had the longest history of enrolling and had completed the most trials. Participants with less than a high school diploma were newest to enrolling and had completed the fewest trials. Participants with a trade or vocational training enrolled in slightly more new trials in 3 years compared to the other groups. Employment^1^: Unemployed participants had the shortest history of enrolling compared to full-time or part-time workers, but they had completed the same number of trials as full-time workers, which was fewer than part-time workers. Over the course of the HVP, unemployed participants completed the most trials, followed by part-time workers, then full-time workers. Income^1^: Participants who earned between $25,000 and $50,000 annually had participated in the most clinical trials over the longest period of time. However, over the course of the HVP, those who earned less than $25,000 annually participated in a greater number of trials than those who earned between $25,000 and $50,000 annually, who in turn enrolled in a greater number of trials than those who earned more than $50,000 annually. “Occupational” Participation: Participants who pursued trials as their full-time job had a longer history of enrolling and had completed more trials. They enrolled in four times as many new clinical trials in 3 years compared to non-occupational participants. ^1^ Employment and income are highly dynamic variables that may not provide accurate depictions of clinical trial participation over time. See Results and limitations sections for more details.

The present study is among the first to describe the clinical trial participation histories of a national convenience sample of people who participate as healthy volunteers in US Phase I clinical trials. These findings contribute to the extant literature on the sociodemographic characteristics of US healthy volunteers by offering more nuanced, longitudinal data about differences in trial enrollment among groups based on sex, race and ethnicity, age, educational attainment, employment status, and household income [1–3]. Although it is sometimes assumed that primarily young university students enroll in paid research [21, 22], our findings further confirm that Phase I participants are overly representative of groups subject to social and economic inequalities in the United States (Fig 1). Specifically, more than two-thirds of our participants were members of a racial and/or ethnic minority group, and we found that compared to non-Hispanic Whites, minorities also had participated in a greater number of Phase I trials and were more likely to still be screening and participating in clinical trials three years later. In particular, Black participants had the longest history of trial enrollment prior to the HVP and enrolled in double the number of new trials during the HVP compared to both Hispanics and Non-Hispanic Whites. As a group, HVP participants also had low educational attainment and were underemployed, including more than 40% who were jobless. Nearly half had an annual income of less than $25,000 at the time of enrollment in the HVP. While participants ranged in age from 18 to 64, the majority were in their 30s and 40s. Fig 2 summarizes our findings of how demographic groups’ Phase I trial history and experience differed at baseline and after following participants for the three years of the HVP.

The overrepresentation of people of color in Phase I trials relative to the US population as depicted in our sample of healthy volunteers is congruent with previous literature that has raised concern about the exploitation of these groups within biomedical research [1, 3]. Because Phase I trial participants are healthy, no therapeutic advantages are possible, unlike in Phase III trials where racial and ethnic minorities continue to be underrepresented [6]. Far from a recruiting success story, then, US minorities ultimately bear greater risks and enjoy fewer benefits from participation in clinical trials. The participation of people of color in Phase I trials also complicates the narrative that these groups are simply distrustful of research and cannot be persuaded to enroll in clinical trials [1]. Thus, the pharmaceutical and contract research industries can do far more to ensure population representativeness of race and ethnicity in all phases of clinical research to reduce disproportionate burdens on minorities within drug development.

At the same time, females accounted for only 26% of HVP participants. The underrepresentation of female healthy volunteers has critical implications for understanding the safety profile of new drugs. There is ample evidence of sex-based differences wherein females experience more numerous and severe adverse reactions from marketed drugs than do males, and Phase I trials may be contributing to the lack of evidence regarding appropriate and safe doses for the sexes [23, 24]. Females’ lower rates of Phase I participation appear to be due largely to clinical trial inclusion–exclusion criteria [25–27]. Yet, limitations on females’ participation also create a disparity regarding income opportunities: males have more opportunity to earn money from Phase I trials compared to females [27].

We also identified important economic-based disparities in our study. At baseline, nearly half of the participants had annual household incomes below $25,000 and were un- or under-employed, meaning they had no work or held part-time jobs. While it is likely not surprising that individuals participating in these financial-incentive-based trials are relatively poor, our data underscore that serial participation is driven by lack of employment opportunity, as well as acute and persistent financial need. In particular, people who were unemployed and people with annual household incomes of less than $25,000 completed the most clinical trials in three years, and both of these groups were likely to still be screening and enrolling in trials at the end of the HVP compared to part-time and full-time workers and compared to individuals who had annual household incomes of over $25,000. Even part-time workers enrolled in more trials than full-time workers and had considerable persistence in screening for new trials. Part-time workers also more actively sought trials to supplement their incomes, which were likely much lower relative to full-time workers, even when they screened but did not enroll in new trials.

As previously reported in the literature, wages that Phase I participants earn are insufficient to change their overall financial circumstances [28, 29]. From enrolling in one to two clinical trials per year, healthy volunteers can expect to earn ~$4,000, and even with extensive screening for new studies, it is unusual for someone to earn more than $10,000 annually from trial enrollment [15]. Thus, participation in the clinical trial enterprise should not be seen as an endeavor that will lead individuals to financial solvency [3, 30, 31]. Indeed, because any single Phase I trial is unlikely to transform healthy individuals’ economic stability in the longer term, many enroll simply to mitigate against financial crisis [32]. This trend raises questions about the voluntariness of their “decision” to pursue clinical trials instead of more traditional forms of work [33]. Fortunately for many of the participants in our sample, many found full-time work over the course of the three years we followed them, and about one-third of our sample reported more annual income by the end of the HVP. These findings indicate that many healthy volunteers do not want to rely on clinical trials for income, and many are pursuing opportunities for education or work to improve their financial situation [14, 29, 30], which means, for some participants, clinical trials can serve as a temporary financial safety net [3, 28, 32].

Our study also confirms the predominance of serial participants in Phase I trials. Individuals in our sample had considerable experience enrolling in trials, with only about 20% of participants in their first trial when recruited for the HVP. More than 50% of the sample had enrolled in five or more trials at baseline, and by the end of our study, nearly three-quarters of the sample had completed that many trials. Older participants also had, on average, a longer history of enrolling in trials. This, again, points to the serial nature of participation in which people are accruing experience in trials over time rather than individuals finding and enrolling in Phase I trials at different periods in their lives.

**Fig 3:**
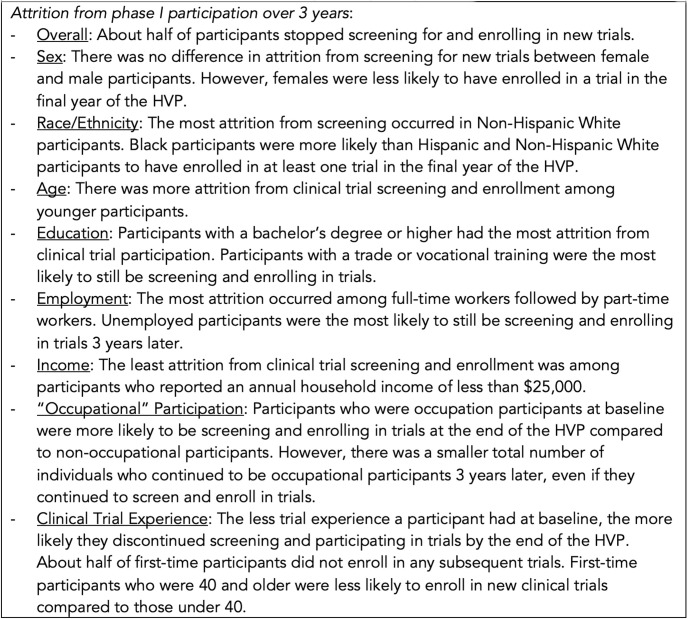
Narrative summary of attrition from phase I participation. *Attrition from phase I participation over 3 years*: Overall: About half of participants stopped screening for and enrolling in new trials. Sex: There was no difference in attrition from screening for new trials between female and male participants. However, females were less likely to have enrolled in a trial in the final year of the HVP. Race/Ethnicity: The most attrition from screening occurred in Non-Hispanic White participants. Black participants were more likely than Hispanic and Non-Hispanic White participants to have enrolled in at least one trial in the final year of the HVP. Age: There was more attrition from clinical trial screening and enrollment among younger participants. Education: Participants with a bachelor’s degree or higher had the most attrition from clinical trial participation. Participants with a trade or vocational training were the most likely to still be screening and enrolling in trials. Employment: The most attrition occurred among full-time workers followed by part-time workers. Unemployed participants were the most likely to still be screening and enrolling in trials 3 years later. Income: The least attrition from clinical trial screening and enrollment was among participants who reported an annual household income of less than $25,000. “Occupational” Participation: Participants who were occupation participants at baseline were more likely to be screening and enrolling in trials at the end of the HVP compared to non-occupational participants. However, there was a smaller total number of individuals who continued to be occupational participants 3 years later, even if they continued to screen and enroll in trials. Clinical Trial Experience: The less trial experience a participant had at baseline, the more likely they discontinued screening and participating in trials by the end of the HVP. About half of first-time participants did not enroll in any subsequent trials. First-time participants who were 40 and older were less likely to enroll in new clinical trials compared to those under 40.

Our longitudinal data also provide some information about the longevity of serial participation. For the sample as a whole, participants enrolled in an average of three new trials over the three years of the HVP. Importantly, we found that the more Phase I trial experience someone had, the more likely it was that they continued enrolling in new trials. The sharpest decline in participation occurred among first-time participants, of whom only 17% were still screening for new trials three years later, whereas two-thirds of people who had already enrolled in more than 10 trials at baseline were still screening for new trials. Examining the sample as a whole, only about half of our participants actively continued to screen for and enroll in trials in the final year of the HVP (Fig 3). The distinction between screening and enrolling is important because our data suggest that considerably more individuals continue to look for new trials and consider enrolling than those who actually complete trials. In particular, the largest gap between screening and participation rates was seen for females, Blacks, Hispanics, 18-29-year-olds, people with less than a high school education, part-time workers, and people who earned less than $25,000 annually. A host of reasons could explain this discrepancy including individuals’ difficulty qualifying for trials (particularly for females), accommodating the trial schedule (particularly for part-time workers), or managing transportation to the clinic location for all study visits (particularly for low-income populations). Additionally, after screening and receiving information in the consent form, concerns about trial risks or inconvenience might also make some people choose to decline participation [34]. More sustained research could explain why certain groups screen for trials at much higher rates than they enroll.

Importantly, the groups most likely to stop participating in trials were the most privileged in our sample: non-Hispanic Whites, 18-29-year-olds, people with a bachelor’s or other advanced degree, full-time workers, and individuals whose household income was greater than $50,000 annually. Patterns in attrition from clinical trials underscore the extent to which social inequalities drive serial participation and trial enrollment. The lower rates of attrition among racial/ethnic minorities and those participants with limited educational attainment may be explained by a higher chance of precarious employment for these groups, who are less likely to find and maintain secure, well-paying jobs over time, relative to non-Hispanic Whites and those with advanced degrees [35]. Thus, attrition from or sustained enrollment in Phase I trials over time is likely to be influenced by these broader trends in employment and involvement in low-wage work.

Further, the literature on Phase I trials often focuses on so-called professional guinea pigs, assuming they are the majority of healthy volunteers who participate [16, 17]. Yet, prior to our study, there had never been a count of what we prefer to call occupational participants relative to other healthy volunteers who also might enroll serially but do not pursue clinical trials full time. About one-third of HVP participants at baseline were designated occupational participants based on their self-report. These individuals had, on average, enrolled in many more Phase I trials than the other participants. They were also the group most likely to still be participating at the end of the HVP. However, only 1 out of 3 were still occupational participants three years later despite their continued involvement with trials. Indeed, the majority of healthy volunteers throughout our study did not pursue clinical trials as a profession.

Our study has several limitations that are important to discuss. The variables we included are all based on data that were self-reported in a questionnaire. Variables such as household income can be unreliable, especially when participants have nonstandard ways of determining whose and what income count toward these totals [36]. In addition, household income and employment status are difficult variables to use in longitudinal analyses of clinical trial activity because both are subject to significant volatility over time, particularly for populations that face significant structural impediments to securing and maintaining well-paid jobs [37, 38]. Other variables such as those related to clinical trial history at baseline rely on participants’ memories, so the true number of clinical trials or length of their trial involvement might be smaller or larger depending on the participant. Additionally, our modest sample size limits the precision of our estimates and the ability to statistically compare our estimates across various demographic strata. While we did not have a random sample of healthy volunteers, having had to rely on convenience sampling, there is no reason to believe that our sample is unique from the US Phase I participant population as a whole.

Our study has several strengths over existing research studies. We are among the first studies to identify and describe a population of healthy volunteers participating in US Phase I trials and to investigate the clinical trial activity of that sample over time. We have been able to show that not only are racial and ethnic minority groups more likely to enroll in Phase I trials, but they are also more likely than non-Hispanic Whites to participate in a greater number of these trials and over a greater number of years. Our findings also confirm the extent to which females are underrepresented in Phase I trials compared to males. Additionally, our study provides insight into the prevalence of occupational research participants who regularly enroll in Phase I trials, indicating that they are an important segment of this population but nonetheless are not the majority of individuals who enroll. Overall, our findings help to situate clinical trial participation as an economic activity that appears to be more important to the most socially disadvantaged groups in the US.

## Conclusions

Racial and ethnic minorities continue to participate as healthy volunteers in US Phase I clinical trials in disproportionate number compared to Non-Hispanic Whites. Additionally, people of color are more likely to rely on Phase I trials as a critical income source. Conversely, women are underrepresented in Phase I trials, which has negative economic implications for healthy females and creates potential health risks for female patients when pharmaceuticals are eventually approved for the market. Regarding other demographic variables such as age, employment status, and household income, more disadvantaged groups are likelier to continue their clinical trial participation over the long term. Even if clinical trials do not provide significant income, they serve an important stopgap measure for individuals and families struggling to make ends meet. These patterns of Phase I trial enrollment, especially long-term serial participation, suggest that the current system of testing the safety of new pharmaceuticals profoundly depends on social inequalities to facilitate recruitment and enrollment for these trials.
