# Correction: Healthy volunteers in US phase I clinical trials: Sociodemographic characteristics and participation over time

**DOI:** 10.1371/journal.pone.0263362

**Published:** 2022-01-26

**Authors:** The PLOS ONE Staff

In Table 1, the values for Sex, % female and Racial/ethnic minority, % appear in the incorrect rows. Please see the correct Table 1 here. The publisher apologizes for the errors.

**Table 1:** Characteristics of HVP study participants (enrolled v. retained).

	Baseline (n = 178)	3-years later (n = 166)
	N (%)	N (%)
**Sex, % female**	47 (26)	45 (27)
**Age group**		
18–29	40 (22)	23 (14)
30–39	58 (33)	45 (27)
40–49	54 (30)	51 (31)
50+	26 (15)	47 (28)
**Race/ethnicity** ^**a**^		
Asian or Asian American	6 (3)	6 (4)
American Indian or Alaskan Native	2 (1)	2 (1)
Black or African American	72 (41)	66 (40)
Native Hawaiian or Pacific Islander	2 (1)	2 (1)
White	83 (47)	80 (48)
More than one race	13 (7)	10 (6)
Hispanic	38 (21)	33 (20)
**Racial/ethnic minority, %**	121 (68)	111 (67)
**Educational attainment**		
Less than high school	12 (7)	12 (7)
High school/GED	37 (21)	29 (18)
Some college	52 (29)	40 (24)
Trade or vocational training	19 (11)	23 (14)
Associate’s (2-year) degree	21 (12)	20 (12)
Bachelor’s (4-year) degree	32 (18)	37 (22)
Graduate degree	5 (3)	5 (3)
**Employment status** ^**b**^		
Full-time, including business owners	45 (25)	76 (46)
Part-time, including seasonal & gig work	60 (34)	47 (28)
Not employed or retired	73 (41)	43 (26)
**Household income** ^**c**^		
Less than $10,000	30 (17)	20 (12)
$10,000–$24,999	52 (29)	36 (22)
$25,000–$49,999	71 (40)	64 (39)
$50,000–$74,999	13 (7)	26 (16)
$75,000–$99,999	7 (4)	6 (4)
≥ $100,000	4 (2)	11 (7)

^a^ The category Hispanic includes all racial groups, of which we have those in our sample who identified as White, Black, more than one race, American Indian, and Native Hawaiian/Pacific Islander.

^b^ These data are based on consolidated definitions of each employment category that we used to standardize self-reported data from participants.

^c^ Household income was not reported by one participant at baseline and three at the end of the HVP.
